# 

**Published:** 1885-01

**Authors:** 


VOL. VI.] TWO DOLLARS A YEAR. SINGLE COPIES 60 CENTS. [No. 1.

### THE JOURNAL OF COMPARATIVE MEDICINE AND SURGERY.

1131
LIBRARY INDEXED S.G.O.

### A QUARTERLY JOURNAL OF THE ANATOMY, PATHOLOGY AND THERAPEUTICS OF THE LOWER ANIMALS.

*Edited by* { W. A. CONKLIN, PH.D.

F. S. BILLINGS, D.V.S.

Entered New York Post Office as second class matter.

### JANUARY, 1885—CONTENTS.

WILLIAM R. JENKINS,
VETERINARY PUBLISHER, BOOKSELLER AND PRINTER,
850 Sixth Ave., Cor. 48th Street,
NEW YORK.
LONDON: BAILLIÈRE, TINDALL & COX.

